# Synergistic corrosion effects of magnetite and microorganisms: microbial community dependency

**DOI:** 10.1007/s00253-024-13086-6

**Published:** 2024-03-05

**Authors:** Maria A. Diaz-Mateus, Laura L. Machuca, Hanan Farhat, Silvia J. Salgar-Chaparro

**Affiliations:** 1 Curtin Corrosion Centre, WA School of Mines: Minerals, Energy and Chemical Engineering, Curtin University, Bentley, WA Australia; 2 WA School of Mines: Minerals, Energy and Chemical Engineering, Curtin University, Bentley, WA Australia; 3 Qatar Environment & Energy Research Institute (QEERI), Doha, Qatar

**Keywords:** Microbiologically influenced corrosion, Acid-producing bacteria, Magnetite, Carbon steel, pH, Deposits

## Abstract

**Abstract:**

The synergistic corrosion effect of acid-producing bacteria (APB) and magnetite on carbon steel corrosion was assessed using two different microbial consortia. A synergistic corrosion effect was observed exclusively with Consortium 2, which was composed of *Enterobacter* sp., *Pseudomonas* sp., and *Tepidibacillus* sp. When Consortium 2 was accompanied by magnetite, uniform corrosion and pitting rates were one-time higher (0.094 mm/year and 0.777 mm/year, respectively) than the sum of the individual corrosion rates promoted by the consortium and deposit separately (0.084 and 0.648 mm/year, respectively). The synergistic corrosion effect observed exclusively with Consortium 2 is attributed to its microbial community structure. Consortium 2 exhibited higher microbial diversity that benefited the metabolic status of the community. Although both consortia induced acidification of the test solution and metal surface through glucose fermentation, heightened activity levels of Consortium 2, along with increased surface roughness caused by magnetite, contributed to the distinct synergistic corrosion effect observed with Consortium 2 and magnetite.

**Key points:**

• *APB and magnetite have a synergistic corrosion effect on carbon steel.*

• *The microbial composition of APB consortia drives the synergistic corrosion effect.*

• *Magnetite increases carbon steel surface roughness.*

## Introduction

Corrosion represents a critical engineering problem that results in millions of dollars in losses to the oil and gas industry annually (Koch et al. 2016). From this total cost, microbiologically influenced corrosion (MIC) accounts for almost 20% of external and 40% of internal corrosion problems in pipelines (Wolodko et al. 2018). Metal deterioration can lead to complete pipe failures that, besides the financial consequences, cause substantial environmental damage and represent high health and safety risks for workers in oil and gas fields (Beech et al. 2014). While industry countermeasures like pigging, chemical treatment, coatings and ultraviolet (UV) irradiation are implemented against MIC (Little et al. 2020), the complex nature of this corrosion mechanism makes it very challenging to control. In the last years, MIC research has proven that environmental factors such as salinity (Zhou et al. 2021), temperature (Li et al. 2017) and pH (Ismail et al. 2014) affect the behaviour of microbial communities and, subsequently, corrosion rates. However, a limited understanding remains of how deposits along pipelines influence MIC, partially contributing to the industry’s struggles in controlling it.

While different studies have provided evidence of the corrosion effect resulting from the conjoint action of deposits and microorganisms on carbon steel (Machuca et al. 2017; Li et al. 2019; Liu and Cheng 2020), they have predominantly focussed on sulphate-reducing bacteria (SRB) as most of MIC laboratory investigations. This emphasis may have fostered the misconception that other physiological groups implicated in carbon steel corrosion, such as acid-producing bacteria (APB), play only a minor role in MIC. Contrarily, APB serves as a key example of chemical MIC (CMIC), where corrosion is caused by the corrosive metabolites produced by the microorganisms during their metabolic activities (Usher et al. 2014; Kryachko and Hemmingsen 2017). The majority of organic acids released by APB as a consequence of the organic sources’ oxidation exist primarily in their un-dissociated molecular form and contribute to metal corrosion by accelerating the cathodic reaction (reduction of hydrogen ions) (Tran et al. 2014), a thermodynamically favourable redox reaction proven capable of corrosion rates as high as 10 mm/year (Gu and Galicia 2012). The presence of organic acids in produced water has represented a concern for the integrity of mild steel pipes in the oil and gas industry for years, leading to extensive research in this area (George et al. 2004; Zhu et al. 2011; Gu 2014; Tran et al. 2014). However, these studies often overlook the biotic source of organic acids, and abiotic substitutes fail in replicating metal deterioration induced by microbial biofilms (Beech et al. 2005).

The synergistic corrosion effect between deposits and bacteria can be defined as the positive interaction between the corrosion promoted by bacteria and the corrosion promoted by deposits which leads to corrosion rates that go beyond what would be achieved by deposits or bacteria independently. To the best of the author’s knowledge, this phenomenon has only been explored by Yang et al. (2022). The authors found that the combined presence of a silica sand deposit and *Desulfovibrio desulfuricans* led to a corrosion rate of 0.035 mm/year, a corrosion rate two times higher than that which would be obtained if the corrosion rates promoted by *Desulfovibrio desulfuricans* and the deposit were added separately (0.018 mm/year). While Yang’s study offers valuable insights into the synergistic corrosion effect of bacteria and deposits on carbon steel, its limitation to a single monoculture of SRB and acid-washed sand raises questions about whether the same trend will be observed in the presence of different microbial taxa and varied deposit types.

Magnetite is a corrosion product frequently found in CO_2_ and H_2_S environments and also prevalent in ruptured pipeline analyses (De Marco et al. 2005; Gao et al. 2018; Bruijnen et al. 2020; Owen et al. 2023). The semi-conductive nature of magnetite provides it with the ability to initiate galvanic corrosion when unevenly distributed in metal surfaces; for this reason, it represents a thread for the oil and gas systems (Song et al. 2018). Recently, microbiology research has proven that magnetite should also be considered a threat to the oil and gas systems because it has been identified as an EET facilitator (Jin et al. 2023; Xu et al. 2023). EET plays a key role in a microbial corrosion mechanism known as electrical MIC (EMIC), where microorganisms directly adsorb electrons from elemental metallic iron, using it as their electron donor. EMIC has been identified as the cause of pit depths as high as 17 μm in carbon steel after a 7-day incubation period (Jia et al. 2017).

The substantial corrosion caused by microorganisms and deposits is commonly attributed to the build-up of corrosive metabolites beneath the deposit (Roberge 2005). Based on these, our hypothesis posits that the acidic metabolic sub-products produced by APB, irrespective of the specific microbial taxa present in the community, are likely to accumulate at the deposit-metal interface, resulting in higher corrosion rates than what would be obtained if the corrosion rates promoted by the consortium alone and the magnetite alone were added separately. This study aims to simulate pipeline systems with zero sulphate concentrations in the production fluids, where nitrate injection to control SRB activity is not implemented. Furthermore, the synergistic corrosion effect of deposits and APB will be evaluated using magnetite as a deposit, given its potential corrosiveness in both biotic and abiotic conditions. Understanding the conjoint action of deposits and microorganisms is critical for developing efficient mitigation and prevention strategies for under-deposit corrosion (UDC) and MIC in the oil and gas industry.

## Materials and methods

### Acid-producing consortia recovery and growth conditions

The two microbial consortia used in this study were recovered from oilfield sand samples collected at two different points of a Western Australian oil production facility (Diaz-Mateus et al. 2023). The two microbial consortia were established from direct sand sample injection in the selective culture media Phenol Red Dextrose Broth for APB (International 2014) with salinity adjusted to mimic the salinity of the production water in contact with sand. Culture media for APB recovery was prepared according to the guidelines and composition described in the standard test method NACE TM0194 under a gas atmosphere of 99.9% N_2_ and served in Hungate tubes. Culture media was inoculated with 1 g of sand and incubated at 40 °C for 20 days. Culture media that exhibited acid production was transferred to fresh liquid media to establish the experimental Consortium 1 and 2.

### Assessment of the synergistic effects of magnetite and acid-producing bacteria on the corrosion of carbon steel

#### Sample preparation

Carbon steel 1030 grade coupons were laser-cut with the dimensions of 12 mm × 22 mm × 9 mm and electro-coated with epoxy resin (Powercron 6000CX, PPG Industrial coatings) to limit the working surface area to one side per sample (2.6 cm^2^). The working surface area was wet-ground through a series of silicon carbide papers to achieve a 600-grit finish. The elemental composition of the carbon steel, determined by atomic emission spectroscopy, is given in Table S1. All samples were degreased with acetone, washed with ethanol and dried with nitrogen gas. Before the corrosion test, the metal coupons were weighted and sterilized by UV radiation for 15 min. Subsequently, 3.6 g of magnetite powders (Sigma Aldrich, Fe_3_O_4_), previously sterilized by autoclaving at 134 °C and 208 kPa for 3 min, was placed on the exposed surface of the metal coupons.

The sterile carbon steel samples were placed in sterile custom-made 3D-printed epoxy boxes to control the diameter and height of the simulated magnetite deposit and allow the consistent deposition of 10 mm of magnetite on top of the metal samples for all groups of experiments. The mean particle size of Fe_3_O_4_ particles was 4.67 µm, and the specific surface area was 5.41 m^2^/g.

#### Experimental matrix

Six distinct tests were conducted to evaluate the synergistic influence of magnetite and acid-producing bacteria on carbon steel corrosion. The corrosion rates caused by Consortium 1 and Consortium 2 alone and combined with magnetite were measured. Additionally, two abiotic tests were included as controls for the synergistic corrosion evaluation. The experimental matrix and test codes are presented in Table 1.

**Table 1:** Test matrix for corrosion testing

Consortium	Magnetite presence	Code	Description
Consortium 1	Yes	C1	Carbon steel exposed to Consortium 1
	No	C1 + M	Carbon steel exposed to Consortium 1 and magnetite
Consortium 2	Yes	C2	Carbon steel exposed to Consortium 2
	No	C2 + M	Carbon steel exposed to Consortium 2 and magnetite
Controls	Yes	M	Carbon steel exposed to magnetite
	No	B	Carbon steel exposed to test solution

#### Experimental set-up

The corrosion experiments were carried out in a modified 2-L vessel with a working volume of 1.8 L containing a test solution with the following composition: 425 mM NaCl, 10 mM CaCl_2_·2H_2_O, 10.2 mM KCl, 54 mM MgCl_2_·6 H_2_O, 0.09 mM SrCl·6H_2_O, 10 mM D-glucose, 1.3 g L^−1^ casamino acids (Bacto™) and 1 L of ultrapure water (Milli-Q system). The solution pH was adjusted to 7.2 ± 0.2 with a sodium hydroxide solution (2 M). The test temperature was set at 40 ºC, and gentle agitation (180 rpm) was achieved using a stirring digital hotplate (IKA RTC) under thermocouple control. N_2_ gas flow was maintained for the 15 days of testing to keep anaerobic conditions inside the reactors. Biotic reactors were inoculated with the respective microbial consortium in a concentration of 3.2 × 10^6^ cells/mL, and microbial activity was maintained by continuous solution replenishment (40% of the reactor’s total volume daily) throughout the experimental period.

#### Corrosion measurements

After 15 days of testing, magnetite deposits on triplicate coupons from each tested condition were gently washed away with N_2_-saturated ultrapure water to reveal the extent of corrosion. Subsequently, the corrosion products on the exposed surface were removed by ultrasonically cleaning the coupons for 1 min in Clarke’s solution, as described in the ASTM G1-03 Standard (ASTM 2017). Afterwards, the weight of the cleaned metal samples was measured using a high-accuracy mass balance (Mettler Toledo, ME204), and general corrosion rates in mm/year were estimated based on the weight loss and surface area of the metal samples (ASTM 2017).

Exposed surfaces of the triplicate coupons were also analysed using a 3D surface profilometer (Solarius™, SolarScan) with a spot size of 10–100 µm and resolution of 0.2 µm to find the maximum intrusion depth on each coupon. The pitting rate was estimated by dividing the deepest point (mm) found in each condition by the exposure time in days, as described in the NACE SP0775 standard practice (NACE 2013).

#### Total iron and ferrous iron concentration

As an indirect corrosion measurement along the 15-day test, total iron and ferrous iron concentrations in the bulk solution in each condition were measured using a VIS spectrophotometer instrument (Hach™ DR3900) every 3 days. Ferrous iron (Fe^2+^) and total iron (FeT) concentrations were measured by the 1,10-phenanthroline method and USEPA FerroVer® method, respectively, following the manufacturer’s instructions.

#### Surface analysis

A field emission scanning electron microscope (FESEM) (TESCAN CLARA) was used to examine the metal surface upon metal sample cleaning. Surface roughness analyses were conducted using a 3D surface profilometer (Solarius™, SolarScan). For this, three (3) coupons for each condition were analysed.

#### Metal surface pH measurements

A combination flat surface pH meter (Thermo Scientific™, Orion 8135BN) was used to measure the metal surface pH of deposited and non-deposited samples at the end of the immersion period.

### Microbiological analysis of the microbial communities at the end of the corrosion tests

#### Microbial cell concentration

The number of viable sessile cells developed on each biotic scenario was determined on triplicate samples by the most probable number (MPN) method, using the same test solution of the corrosion test (see “Microbial activity estimation”). Sessile microorganisms were detached from the coupons and magnetite as follows: Bare coupons (C1 and C2) were immersed in Falcon tubes containing 10 mL of sterile phosphate-buffered saline (PBS) solution with Tween 20 (0.1% w/v final concentration). Magnetite was separated from the metal sample in C1 + M and C2 + M samples with a sterile scalpel blade, and the sections with the metal sample were placed in Falcon tubes containing 10 mL of sterile PBS solution with Tween 20 (0.1% w/v final concentration) for microbial cell counts. Sessile cells were recovered in the PBS solution after full-speed vortexing for 10 s in the Falcon tubes containing the samples. This was followed by a 2-min sonication in cycles of 15 s on and 10 s off in ice as described elsewhere (Salgar-Chaparro et al. 2020).

The number of viable APB was determined by following the serial dilution method described in the standard test method NACE TM0194 (NACE 2014). One millilitre of the PBS solution containing the microbial cells was inoculated in a glass vial with 9 mL of culture media and diluted 10 times. Then, vials were incubated at 40 °C for 20 days. Bacteria growth was positive upon a noticeable change in the medium colour and turbidity. The concentration of microorganisms in each sample was calculated using the MPN 3-tube standard table (Da Silva et al. 2018).

#### Microbial activity estimation

The microbial activity levels and physiological state of the sessile communities at the end of the 15-day immersion test were determined by measuring the concentrations of adenosine triphosphate (ATP), adenosine diphosphate (ADP) and adenosine monophosphate (AMP) of each sample using the AXP assay and the Quench-Gone Organic Modified (QGO–M) test kit (LuminUltra™) The concentration of the adenosine nucleotides ATP, ADP and AMP contained in the sessile cells in the different corrosion scenarios was determined by a luminescent substrate-enzyme reaction with luciferin-luciferase; the reaction was read in a PhotonMaster luminometer (LuminUltra™, PhotonMaster) following the manufacturer’s instructions. The adenylate energy charge (AEC) (ratio of ATP, ADP and AMP) was then calculated according to the following equation:

$$AEC=(ATP+0.5ADP)/(ATP+ADP+AMP)$$

#### Microbial taxonomic profiles

16s rRNA gene amplicon sequencing from the DNA and RNA molecule was carried out to characterize the total and active microbial taxa associated with each corrosion test. For this, the PBS containing the sessile cells from the bare carbon steel and magnetite-metal interphase was centrifuged at 15,000 × g for 5 min at 4 °C to pellet the sample, then, DNA and RNA were extracted using Norgen DNA/RNA/Protein kit as directed by the manufacturer (Norgen Biotek Corp). Nucleic acid concentration and quality were verified using a spectrophotometer (Thermo Scientific™, NanoDrop Lite). Following extraction, genomic DNA was digested from the extracted RNA samples using the Turbo DNA-free kit (Invitrogen) according to the manufacturer’s instructions. Afterward, RNA was purified by using an RNeasy MinElute cleanup kit (Qiagen) and transcribed into complementary DNA (cDNA) by the SuperScript IV first-strand synthesis system (Invitrogen), as described previously (Salgar-Chaparro et al. 2020).

The gene‐specific sequences used in this study for Library preparation were selected due to their overall coverage (Klindworth et al. 2013). Forward Primer = 5′ TCGTCGGCAGCGTCAGATGTGTATAAGAGACAGCCTACGGGNGGCWGCAG and Reverse Primer = 5′ GTCTCGTGGGCTCGGAGATGTGTATAAGAGACAGGACTACHVGGGTATCTAATCC, targeting the V4–V5 regions of the 16S ribosomal RNA gene of bacteria and archaea (Yu et al. 2005), were used. Sequencing was performed by the Marshall Centre at the University of Western Australia (UWA) using next-generation paired-end sequencing on a sequencing instrument (Illumina, MiSeq) in accordance with Illumina’s protocol (Illumina 2011).

A bioinformatics analysis of the metabarcoding samples was performed with the Quantitative Insights Into Microbial Ecology Software (QIIME 2) (Callahan et al. 2016). Data were demultiplexed using the “q2-demux” plugin, trimmed and quality filtered using the “dada2 denoise-paired” plugin. Forward and reverse sequence read lengths were truncated at 280 and 220 positions, respectively, with the plugin “- -p-trunc-len-f 280” and “- -p-trunc-len-r 220”. Taxonomy was assigned to each amplicon sequence variant (AVS) using BLAST (“feature-classifier classify-blast”) against the pre-trained SILVA reference database (v138) (Bokulich et al. 2018). The “qiime tools export” function was used to export into R (v4.2.1) the ASV tables generated by QIIME2 for taxonomic composition analysis. The average of the triplicate samples is the reported relative abundance of amplicon sequence variants (ASVs) in the active and total community.

#### Statistical analysis

The data in this study is based on the average of three separate experimental replicates. To analyse the uniform corrosion and pitting rates in the six experimental scenarios, a statistical analysis was performed using PAST (V4.10). The normality of the data for each variable was tested using the Shapiro–Wilk method. A one-way analysis of variance (ANOVA) with Tukey’s post hoc means separation test was conducted to determine if there were any statistically significant differences between normally distributed variables. The results of the statistical tests were considered significant when the *p* value was equal to or less than 0.05.

## Results

### Corrosion assessment

#### Uniform corrosion rates

**Fig.1 Figure 1 was added as attachment with a change in axe title from mmpy to mm/year:**
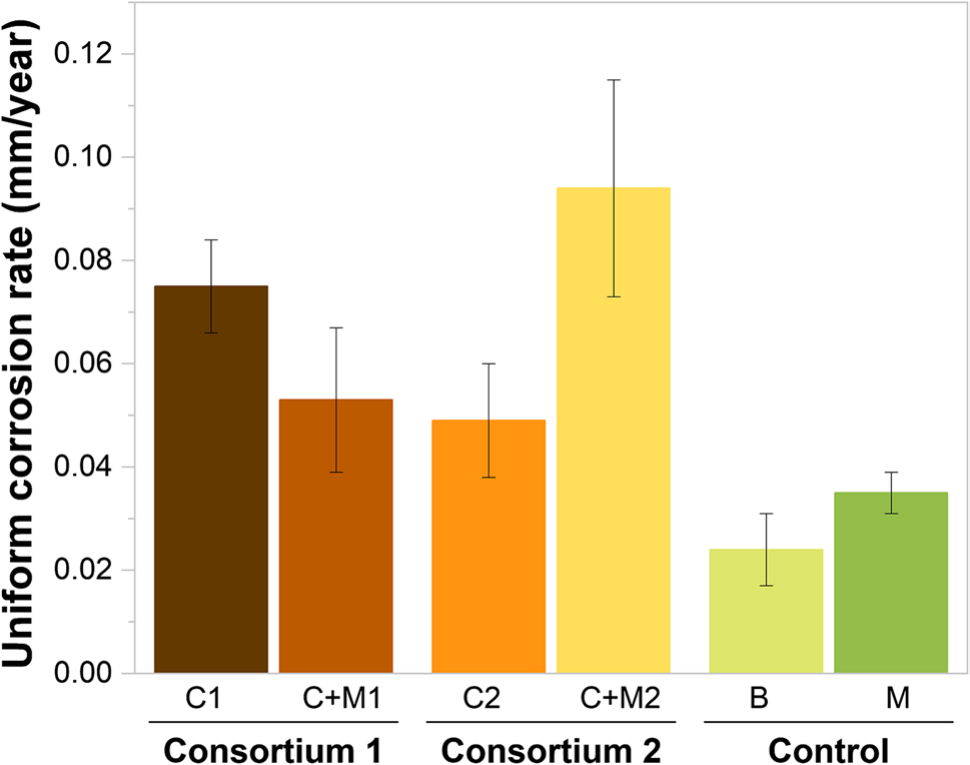
Uniform corrosion rates calculated based on weight loss measurements for blank (B), magnetite (M), Consortium 1 (C1), magnetite + Consortium 1 (C1 + M), Consortium 2 (C2), magnetite + Consortium 2 (C2 + M). Error bars represent standard deviations of the corrosion rates calculated in three independent replicates

Uniform corrosion rates calculated from the mass loss of metal coupons are presented in Fig. 1. In the absence of deposits and APB (B),the corrosion rate measured was 0.024 mm/year. The abiotic magnetite control (M) corrosion rate increased to 0.035 mm/year. These results indicate a uniform corrosion effect on carbon steel by magnetite. In C1 + M, the uniform corrosion rate was 0.053 mm/year. However, when considering the presence of Consortium 1 alone, the corrosion rates were 0.075 mm/year. Since the corrosion caused by the conjoint action of magnetite and the microbial consortium was lower than the corrosion caused by the consortium alone, these results demonstrate that in the presence of C1, there was no synergistic corrosion effect with magnetite. Moreover, there were significant differences between C1 + M and C1, suggesting that the deposit’s presence hindered the pitting rates caused by Consortium 1 (*p* > 0.05, Table S2).

Contrarily, when Consortium 2 was accompanied by magnetite (C2 + M), the corrosion rate reached 0.094 mm/year, while in the presence of Consortium 2 alone, the uniform corrosion rate was 0.049 mm/year. Notably, the combined effect of Consortium 2 and magnetite (C2 + M) yielded a corrosion rate that was one time higher than the sum of the individual corrosion rates promoted by C2 and M (0.084 mm/year). These establish the existence of a synergistic corrosion effect between C2 and magnetite on carbon steel. Furthermore, statistically significant differences were observed between C2 + M and both C2 and M (Table S2), indicating that the combined action of Consortium 2 and magnetite amplified the corrosion rates of magnetite by 2.5 times and the corrosion rates of Consortium 2 by two times.

#### Pitting corrosion

**Fig. 2 Figure 2 was added as attachment with a change in axe title from mmpy to mm/year:**
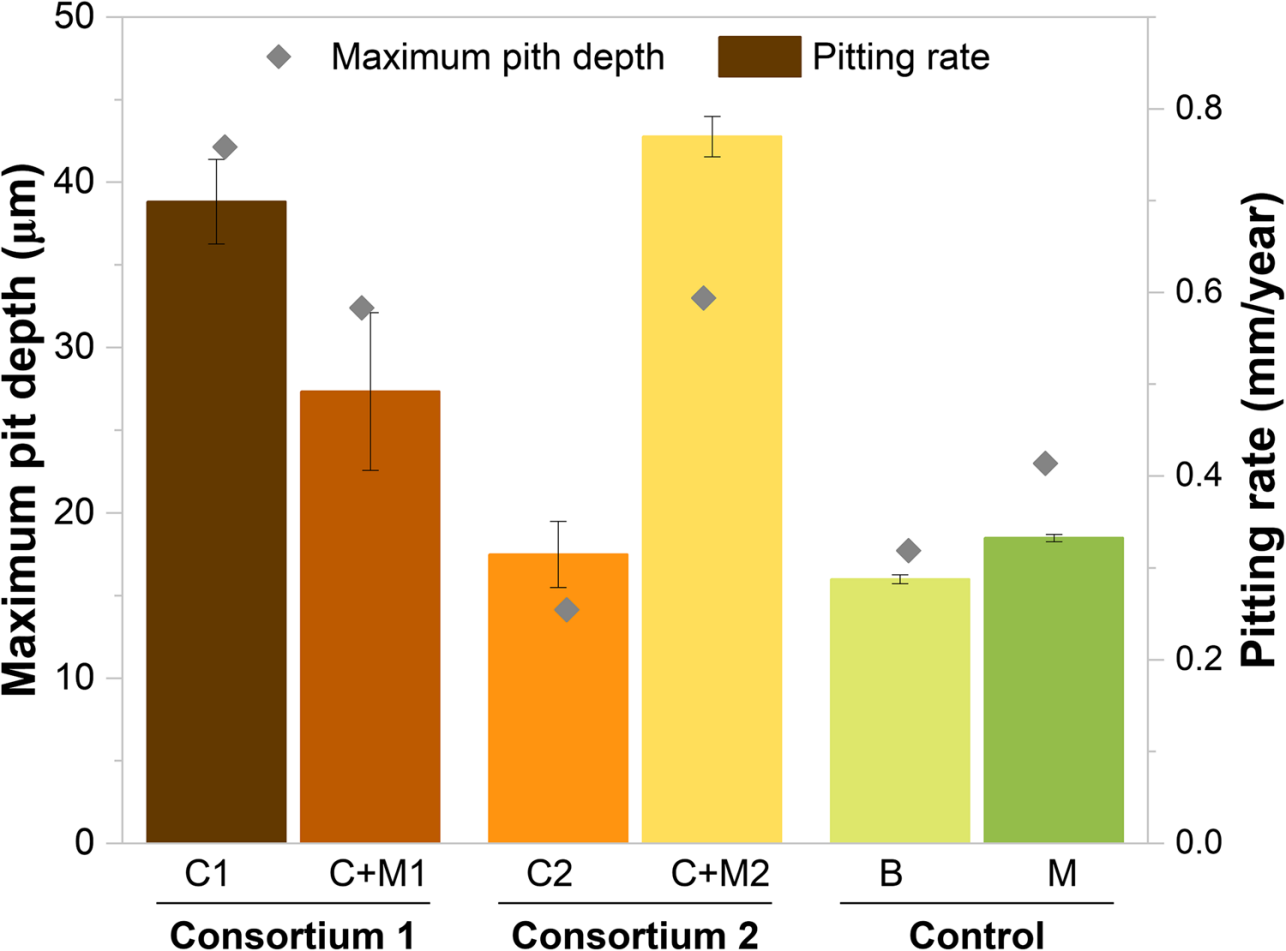
Pitting rates calculated based on the deepest pit on the working surface for Blank (B), magnetite (M), Consortium 1 (C1), magnetite + Consortium 1 (C1 + M), Consortium 2 (C2), magnetite + Consortium 2 (C2 + M). Error bars represent standard deviations of the corrosion rates calculated in three independent replicates

The pitting rates of metal samples, calculated from the maximum pit depth observed in each condition, are displayed in Fig. 2. The pitting rates mirrored the trends seen in uniform corrosion rates (Fig. 1). Higher pitting rates were observed in samples exposed to magnetite alone (0.333 mm/year) compared to the blank (0.288 mm/year). When metal samples were exposed to C1 alone, a pitting rate of 0.699 mm/year was reached; however, when C1 was together with magnetite, a pitting rate 1.4 times lower was measured (0.492 mm/year). These results indicate the absence of a synergistic corrosion effect between C1 and magnetite on carbon steel since the corrosion in C1 + M was two times lower than the sum of the individual corrosion rates caused by C1 and M (1.032 mm/year). However, the statistically significant differences between C1 + M and M (*p* > 0.05, Table S3) indicate that the combined action of Consortium 1 and magnetite amplified the corrosion rates of M by 1.4 times. In addition, the significant differences between C1 + M and C1 suggest that the presence of the magnetite deposit hindered the pitting rates caused by Consortium 1 (*p* > 0.05, Table S3).

On the contrary, pitting rates measured in samples exposed to C2 + M (0.777 mm/year) were one time higher than those obtained when the corrosion rates promoted by C2 alone and M alone were added separately (0.648 mm/year). Moreover, statistically significant differences between the pitting rates of metal samples exposed to C2 + M and those exposed to M, as well as between C2 + M and C2, indicate that the combined action of Consortium 2 and magnetite amplified the corrosion rates of both M and C2 (Fig. 2).

#### Surface analysis

**Fig. 3:**
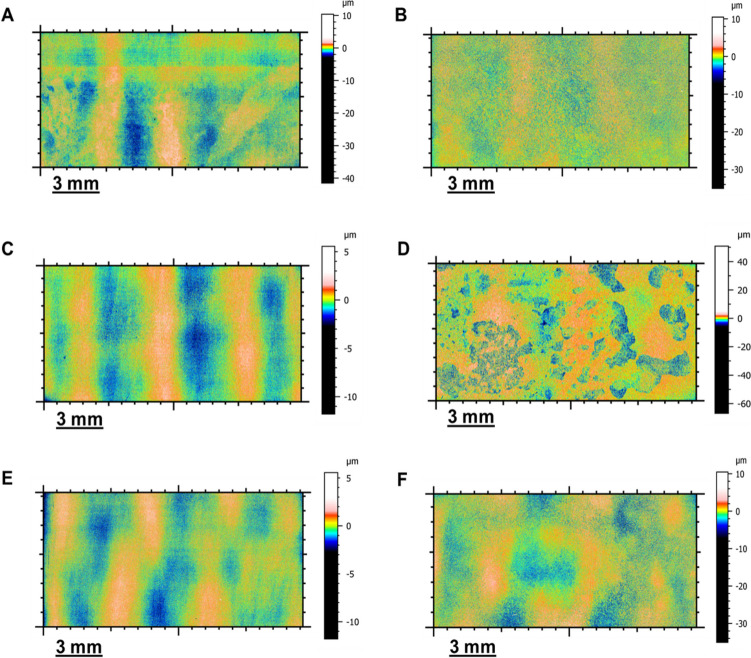
3D optical images of carbon steel exposed to the different corrosion scenarios. **A** Metal sample exposed to Consortium 1 (C1). **B** Metal sample exposed to magnetite + Consortium 1 (C1 + M). **C** Metal samples exposed to Consortium 2 (C2). **D** Metal sample exposed to magnetite + Consortium 2 (C2 + M). **E** Carbon steel exposed to test solution (**B**). **F** Carbon steel exposed to magnetite (M)

**Fig. 4:**
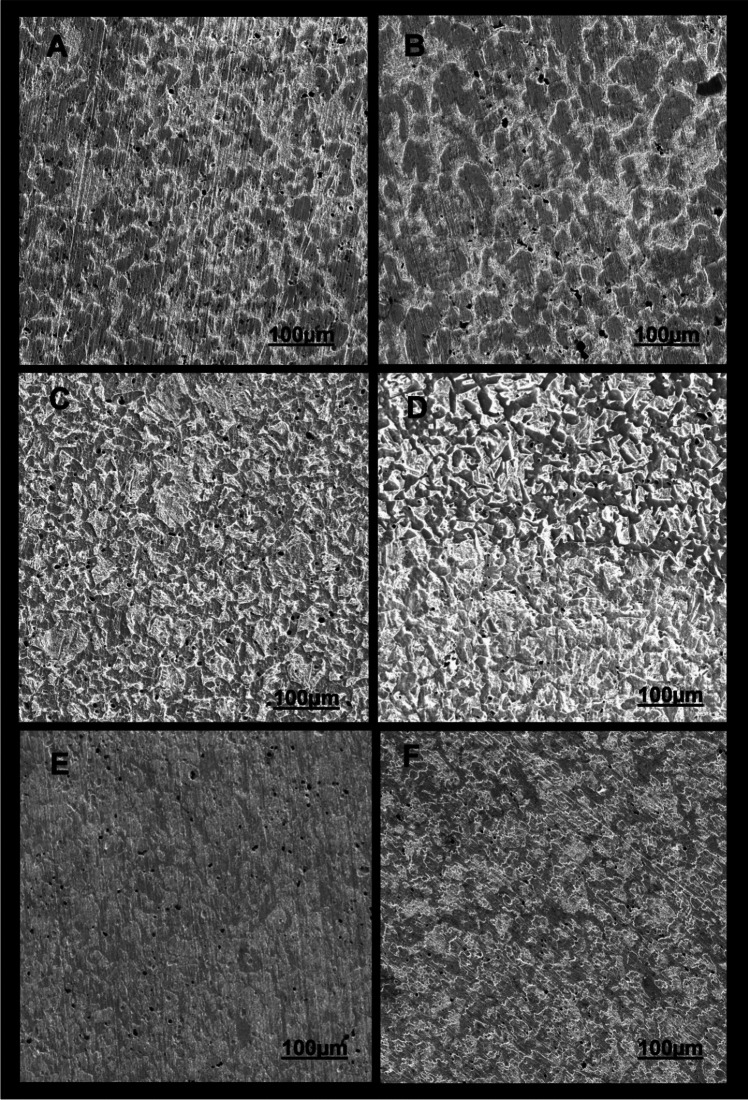
SEM micrographs of carbon steel exposed to the different corrosion scenarios. **A** Metal sample exposed to Consortium 1 (C1). **B** Metal sample exposed to magnetite + Consortium 1 (C1 + M). **C** Metal samples exposed to Consortium 2 (C2). **D** Metal sample exposed to magnetite + Consortium 2 (C2 + M). **E**. Carbon steel exposed to test solution (**B**). **F** Carbon steel exposed to magnetite (M)

Surface profilometry analysis was conducted on the exposed surfaces of carbon steel by step height measurements to find the deepest point on the metal surface. The 3D optical images obtained for one sample from each test condition are summarised in Fig. 3. Generally, the carbon steel surfaces exposed to magnetite (M, C1 + M and C2 + M), whether in biotic or abiotic conditions, appeared rough. In contrast, the carbon steel surfaces exposed to C1, C2 and B (where magnetite was absent) appeared smoother. SEM micrographs presented in Fig. 4 evidence more clearly this phenomena. Although grinding marks were noticeable in the two abiotic controls, M (Fig. 3D) shows higher surface roughness than B (Fig. 4A). These results align with M’s uniform and pitting rates in abiotic conditions (Figs. 1 and 2).

Figure 4 also shows great differences in surface roughness of metals exposed to C1 and C2. Surface roughness seems higher in C2 (Fig. 4E) and C2 + M (Fig. 4F) when compared against the surface roughness of C1 (Figs. 4B) and C1 + M (Fig. 4C). Grinding marks were still noticeable in C1 and C1 + M but were absent in C2 and C2 + M. Furthermore, apparent differences are seen in the presence and absence of magnetite for both consortia, more expansive peak areas in grey (bare metal) and fewer valley areas in white (corroded metal) in C1 + M than in C1. Similarly, results show that the effect of C2 on surface roughness is overall uniform. In contrast, the combined presence of magnetite and C2 led to the formation of localised areas with higher surface roughness than the rest of the surface. Interestingly, in C2 + M, greater pitting (black points) was found in the deeper (dark grey) areas (Figure S1).

The roughness profiles of the coupons exposed to various conditions offered quantitative evidence that supports the qualitative observations made through SEM and 3D profilometry images. These results are presented in Table S4.

#### Fluctuation of dissolved ferrous and total iron concentration

**Fig. 5:**
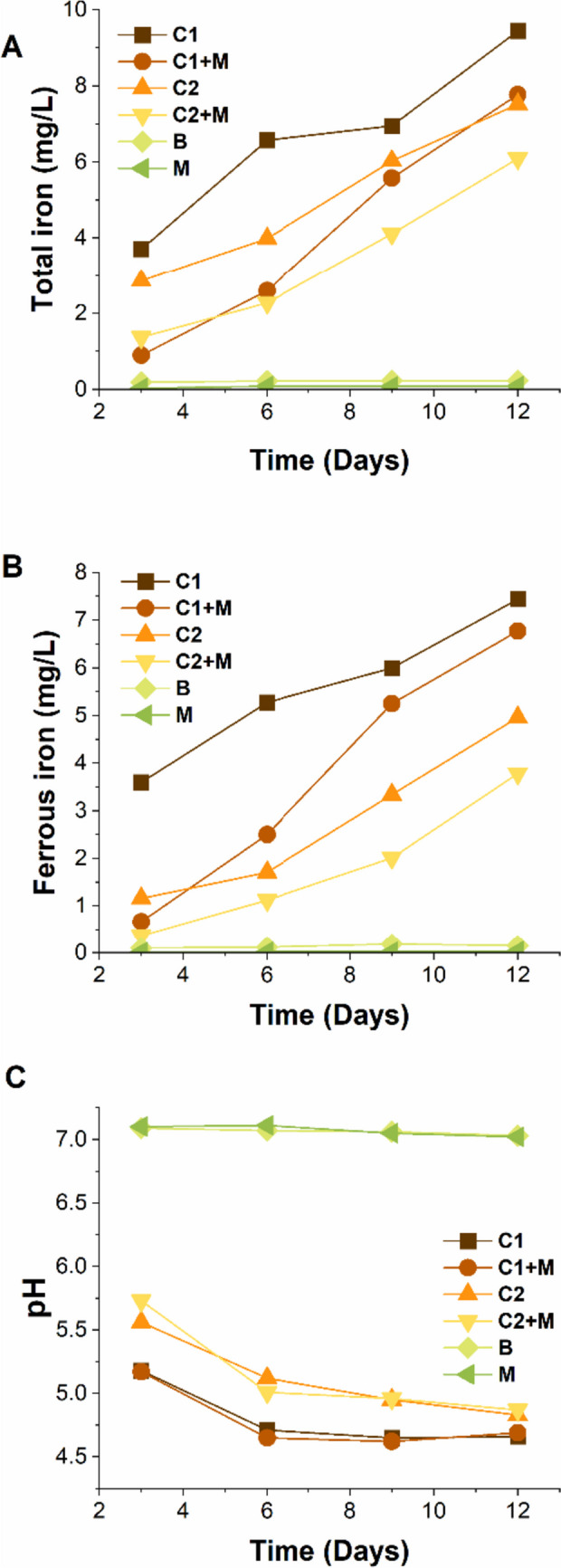
Chemistry monitoring of the test solution over time for the six tested conditions. **A** Dissolved total iron. **B** Dissolved ferrous iron. **C** pH

The trends in dissolved ferrous iron (Fe^2+^), total iron concentrations (FeT) and pH for each of the six corrosion scenarios tested over the immersion period are shown in Fig. 5. In scenarios with the presence of microorganisms, both total iron and ferrous iron concentrations gradually increased from day 1 to day 15. Conversely, total and ferrous iron concentrations remained stable in abiotic control scenarios (B and M). Interestingly, the test solutions in systems involving Consortium 1 (with or without magnetite) showed higher concentrations of iron (both total and ferrous) than those involving Consortium 2. This observation is likely due to the lower pH levels measured on C1 from day 1 to day 12 compared to C2 (see Fig. 5C).

In contrast to the increasing iron concentrations in the test solution of biotic scenarios, the pH levels of C2 and C2 + M decreased from days 1 to 12. Similarly, in C1 and C1 + M, the pH decreased from days 1 to 9, reaching its lowest value, followed by a slight increase until day 12. This pH decline occurred despite the solution being buffered and the initial pH of the test solution being set to 7.2. The decrease in pH indicates that the predominant microbial metabolism in both consortia was the fermentation of glucose, resulting in the production of acids.

#### Local pH measurements

The pH of the metal surface was measured in triplicate at the end of the 15-day immersion period immediately upon retrieval from bioreactors. Results are presented in Table 2. In the metal-deposit interphase, reactors containing C1 exhibited significantly lower pH values (4.95 for C1 and 5.68 for C1 + M) compared to reactors containing C2 (5.19 for C2 and 6.02 for C2 + M) (Table S5). These results are likely associated with the lower pH levels measured in the test solution of C1 and C1 + M during the 15-day immersion period (Fig. 5C) compared to C2 and C2 + M.

**Table 2:** Carbon steel samples average surface pH at the end of the 15-day test in biotic conditions ^a^

	*Consortium 1*		*Consortium 2*	
	C1	C1 + M	C2	C2 + M
*pH*	4.95	5.68	5.19	6.02

^a^Metal surface pH was determined from three measurements from three different samples

Notably, the pH values measured beneath the magnetite deposit were significantly higher than those measured on the metal surface without magnetite for C1 and C2. Additionally, these pH values were higher than the pH of the bulk test solution (Table S5). These results suggest that the magnetite layer limited the mass transfer of acidic species from the bulk solution to the metal-magnetite interphase in deposited samples.

### Microbiological assessment

#### Microbial cell concentration

Enumeration of viable sessile cells attached to the metal on the last day of the test was conducted using the standard serial dilution method. Results are presented in Table 3. In general, higher concentrations of microbial cells were observed in the metal surfaces of tests containing C2 compared to those with C1, regardless of the presence of magnetite. Specifically, 1.10 × 10^6^ cells/cm^2^ and 4.60 × 10^4^ cells/cm^2^ were found in C1 and C1 + M, respectively, whereas C2 and C2 + M exhibited 1.50 × 10^7^ cells/cm^2^ and 2.40 × 10^5^ cells/cm^2^, respectively. In addition, higher concentrations of microbial cells were observed in the absence of magnetite compared to the presence of magnetite at the metal-magnetite interphase, regardless of the microbial consortium under evaluation. These results suggest that magnetite deposits make microbial colonization on metallic surfaces more challenging. In C1 + M and C2 + M, microbial cells face the challenge of traversing from the test solution to the bottom of the deposit. In contrast, in the absence of deposits, the metal surface is readily accessible.

**Table 3:** Cell concentrations of sessile microorganisms in the different experimental conditions

Reactor	Section	MPN (cells/cm^2^) or (cells/g)	95% Confidence limits	
			Lower	Lower
C1	Metal surface	1.10 × 10^6^	1.8	41
C1 + M	Interphase metal/magnetite	4.60 × 10^4^	0.9	20
C2	Metal surface	1.50 × 10^7^	0.37	42
C2 + M	Interphase metal/magnetite	2.40 × 10^5^	0.42	10

#### Microbial activity estimation

**Fig. 6:**
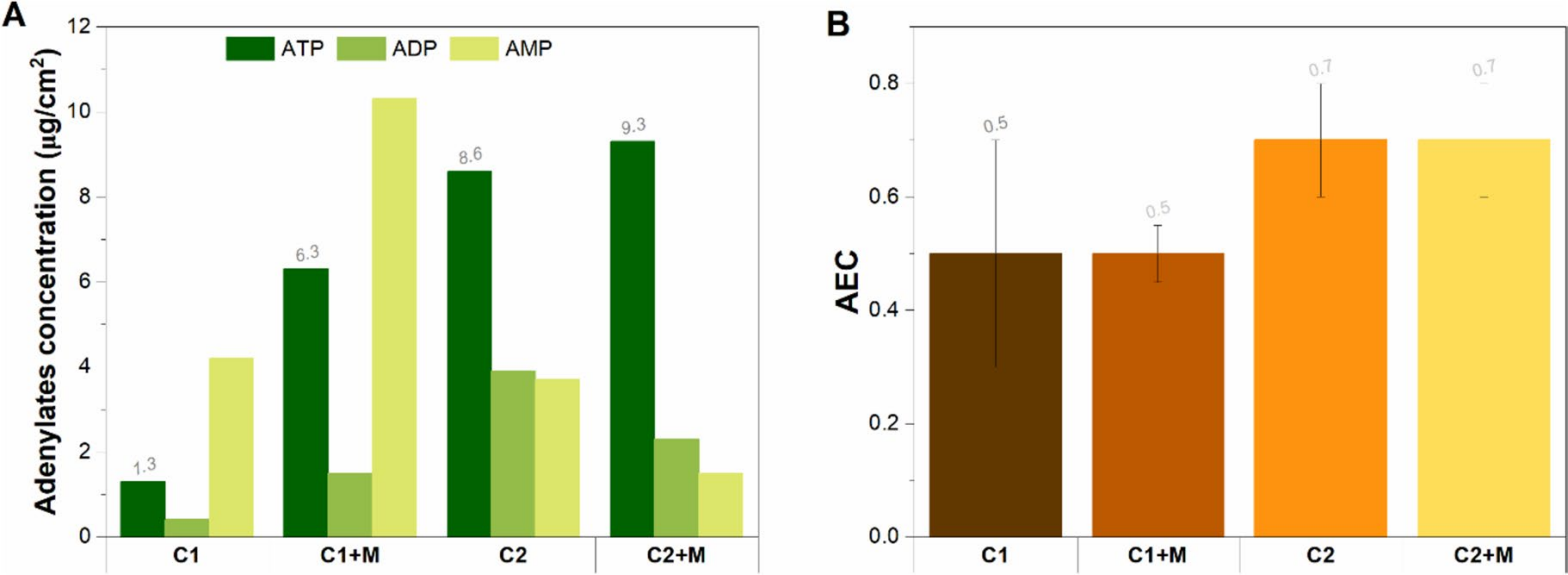
Average adenosine triphosphate concentrations (A) and adenylate energy charges (B) of the sessile communities grown on metal surfaces for Consortium 1 and 2

The adenylate concentration measured on the sessile microorganisms in direct contact with the metal is summarized in Fig. 6A. The results show that Consortium 1 biofilms had lower concentrations of ATP and higher concentrations of AMP than Consortium 2 biofilms, which agrees with the MPN results (Table 3). Statistical analysis revealed a significantly higher number of cells in an active state in C2 than in C1 (*p* > 0.05, Table S6). Furthermore, differences in the ATP proportion between test conditions without deposits and those with deposits remained consistent in both consortia. While the MPN results suggest higher cell concentrations in conditions without magnetite compared to conditions with magnetite, a more significant ATP proportion was observed under the magnetite deposit compared to its absence. This suggests that conditions under the deposit did not favour cell multiplication but a more active metabolism.

The AEC values aimed to assess stress levels and the physiological state of each sessile community are shown in Fig. 6B. AEC values higher than 0.8 typically correspond to metabolically active microbial populations. AEC values ranging between 0.5 and 0.8 indicate stressed but viable populations (i.e., in a stationary growth phase), and AEC values lower than 0.5 correspond to senescent populations (Wiebe and Bancroft 1975). Higher AEC values were observed in tests containing C2 compared to C1, irrespective of the presence of magnetite; however, all AEC values fell within the range of 0.5 and 0.8. This result demonstrates that at the conclusion of the test, the sessile cells of both C1 and C2, whether in the presence or absence of magnetite, were under stress. While not actively growing, they remained viable, as supported by the MPN results (Table 3). This phenomenon is likely linked to the stress imposed by the acidic pH of the environment.

#### Microbial taxonomic profile

**Fig. 7:**
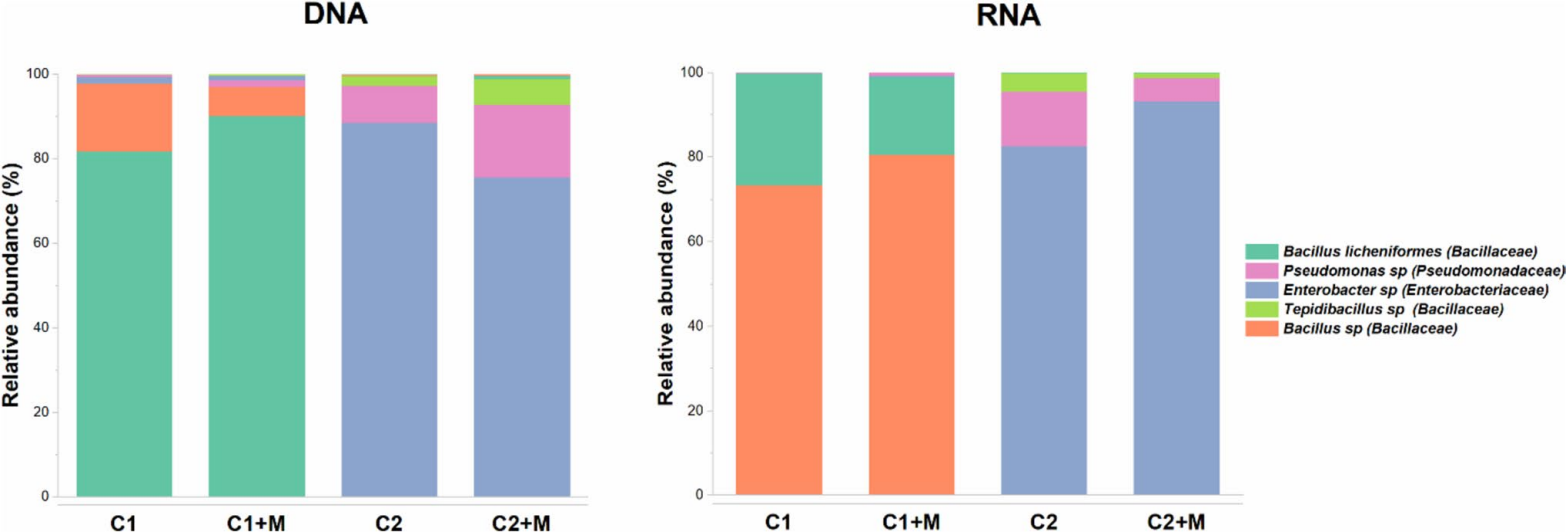
Taxonomic distribution of total (DNA) and active (RNA) microbial populations formed on bare steel (C) and magnetite-deposited carbon steel (C + M) after a 15-day immersion test for Consortium 1 and 2. Each bar represents the results from the three replicates evaluated in each condition

The total and active microbial community within biofilms grown under the different corrosion scenarios was identified through 16S DNA and RNA-based amplicon sequencing. After quality filtering of the raw reads, 2,057,692 high-quality sequences were obtained. These sequences were taxonomically classified into four microbial genera, as shown in Fig. 7. Molecular identification of the microorganisms revealed noticeable differences between the two microbial consortia, primarily concerning the dominant presence of the family *Bacillaceae* in Consortium 1 and *Enterobacteriaceae* in Consortium 2. Differences in the relative abundances of less prevalent microorganisms were also observed between the two consortia. It is important to note that no single genus was significantly active in both consortia. Furthermore, the comparison of the DNA and RNA profiles showed more pronounced differences in the relative abundance of various taxa in the total and active population of Consortium 1 than in Consortium 2, regardless of the presence or absence of magnetite. This discrepancy is because DNA-based analysis considers active, dormant and dead cells, whereas RNA-based analysis solely considers active cells (Salgar-Chaparro and Machuca 2019).

In Consortium 1, the total communities were primarily dominated by *Bacillus licheniformis*, accounting for 81 and 87% of the total community in C1 and C1 + M, respectively. However, this species exhibited lower relative abundances in the active communities, comprising 26% and 18% in C1 and C1 + M, respectively. A similar pattern was observed with *Enterobacter* sp., which constituted 1.9 and 3.5% of the total community in C1 and C1 + M, respectively, but represented only 1.2 and 1% of the active community in C1 and C1 + M, respectively. Conversely, *Bacillus* sp. showed the opposite pattern, with lower relative abundances in the total community and higher abundances in the active community. Interestingly, no discernible differences in microbial composition resulted from the presence of magnetite in either Consortium 1 or Consortium 2.

In contrast, the total and active communities in Consortium 2 were similarly dominated by *Enterobacter* sp., *Pseudomonas* sp. and *Tepidibacillus* sp. For instance, *Enterobacter* sp. accounted for 88% and 75% of the total community in C2 and C2 + M, respectively, and 85 and 73% of the active community in C2 and C2 + M, respectively. The most significant difference between total and active communities in C2 was observed in the decrease of the relative abundances of members of the *Bacillaceae* family in the active community compared to their relative abundances in the total community. *Bacillus* sp. accounted for 1.48 and 0.43% of the total community in C2 and C2 + M, respectively, but its abundance decreased to 0.02 and 0.1% in the active community for C2 and C2 + M, respectively.

## Discussion

Our study investigated the synergistic corrosion effect of APB microorganisms and magnetite on the corrosion of carbon steel. Our primary objective was to assess whether similar results would be obtained regardless of microbial community differences between the two consortia. To achieve this, we compared the uniform corrosion and pitting rates obtained with the two different consortia and magnetite to carbon steel corrosion rates and pitting rates obtained without microorganisms (Blank) and promoted by each microbial consortium alone. Corrosion measurements demonstrated that the microbial community composition drives the synergistic corrosion effects between magnetite and APB. It was shown that the concurrent presence of APB Consortium 1 and magnetite did not exhibit a synergistic corrosion effect. Specifically, the combination of C1 + M exhibited higher corrosion rates than when magnetite was present alone but lower than when C1 was present alone. In contrast, when APB Consortium 2 was combined with magnetite, it led to significantly higher uniform corrosion rates and pitting rates than that which would be obtained if the corrosion rates promoted by the microbial consortium and magnetite were added separately.

In the corrosion tests made with Consortium 1, the higher uniform corrosion and pitting rates in C1 than those in C1 + M agreed with a higher concentration of cells per cm^2^ (Table 3). However, it is known that there is no direct correlation between cell concentration and MIC corrosion kinetics, regardless of the quantification method (Little and Lee 2014; Little et al. 2020). Instead, different studies have found correlations between microbial corrosivity and microbial metabolic activities. For instance, Li et al. (2022) found higher corrosion rates and pit depths in the presence of 5 × 10^6^ cells/mL of *Bacillus licheniformis* engaged in extracellular electron transfer, taking electrons directly from mild steel than in the presence of 1.7 × 10^7^ cells/mL of *Bacillus licheniformis* engaged in acid production. Similarly, Salgar-Chaparro et al. (2022) demonstrated that 1 × 10^9^ cells/cm^2^ of *Shewanella chilikensis* DC57 caused lower pitting rates (0.27 mm/year) when using nitrate as a terminal electron acceptor in anaerobic respiration, compared to 1 × 10^6^ cells/cm^2^ of *Shewanella chilikensis* DC57 when using thiosulphate as terminal electron acceptor (1.04 mm/year). Moreover, the AMP concentrations and AEC ratios measured for the sessile communities in C1 and C1 + M demonstrated that sessile cells of the consortium were under severe stress, with most of them being metabolically inactive, regardless of magnetite’s presence. Under these physiological conditions, sessile microorganisms seem unlikely to influence metal corrosion. Considering the pH levels measured in the bulk solution throughout the immersion period and the metal surface pH at the end of the tests, it can be interpreted that both uniform corrosion rates and pitting rates observed in C1 and C1 + M (higher than abiotic controls) are primarily due to the presence of acidic species in the bulk liquid, resulting from the glucose fermentation by planktonic cells. Nevertheless, ATP concentrations and AEC ratios of sessile cells were measured on the last day of the test; it is likely that during the initial colonization of deposited metal, Consortium 1 was active and could have also promoted carbon steel corrosion. Additional studies with metal samplings and ATP measurements at different time points are required to validate our hypothesis.

Statistical analysis revealed significant differences between the metal surface pH of C1 (4.95) and C1 + M (5.68), as well as between the metal surface pH of C1 and the test solution pH (T1) (4.66) (Table S5). These results suggest that both magnetite and biofilm acted as a diffusion barrier for the acids present in the test solution from reaching the metal in C1 and C1 + M. Moreover, the discrepancies in the ATP proportion and MPN results between test conditions without deposits and those with deposits suggest that the magnetite also hindered diffusion of the carbon source required for cell multiplication. The role of deposits as mass-transfer barriers has been well documented for various deposits, such as calcite (Umoru et al. 2012; Mansoori et al. 2019), iron carbonate scales (Nordsveen et al. 2003), sand (Huang et al. 2010; Pandarinathan et al. 2013), and even magnetite, in the form of passive films (Han et al. 2009; Nieuwoudt et al. 2011; Song et al. 2018). Their limited mass transfer effect arises from the formation of a porous layer that blocks the electrolyte pathway from the bulk liquid to the steel surface. Similarly, the role of biofilms in protecting against metal corrosion has been described by different authors (Sowards and Mansfield 2014; Li et al. 2021). Biofilms can prevent metal corrosion by forming a protective layer that acts as a diffusion barrier to the corrosive substances (Kip and van Veen 2015). The higher uniform and pitting rates observed in C1 compared to C1 + M are likely due to the protective layer imposed by magnetite being thicker and more uniform than the layer set by the biofilm.

Contrary to the corrosion trends evidenced with Consortium 1, a synergistic effect of magnetite and APB on carbon steel corrosion was observed when Consortium 2 was used. These differences were attributed to the dissimilarities in the microbial community structure and composition of sessile populations developed by the two consortia. While Consortium 1 comprised two microbial species belonging to the *Bacillus* genus, Consortium 2 comprised three different genera: *Tepidibacillus*, *Enterobacter* and *Pseudomonas*. Despite various authors have identified *Bacillus* species as causative microorganisms in MIC on carbon steel, the prevailing corrosion mechanism proposed is iron oxidation coupled with nitrate reduction (Xu et al. 2013; Li et al. 2022). Moreover, in a study conducted by Lin and Madida (2015), the corrosiveness of 11 *Bacillus* isolates was explored under different nutrient conditions. The results revealed significantly higher corrosion rates in the presence of nitrate supplementation compared to the addition of sucrose, fructose and galactose. Consequently, it is plausible that the maximum corrosiveness of the two *Bacillus* species constituting C1 was not fully realized under our tested conditions, where glucose fermentation was the predominant process.

Moreover, diverse microbial communities are recognized for their greater ability to survive in different environments when compared to single-species biofilms, as the complex interspecies interactions in mixed-species bacterial biofilms enhance the overall function of the biofilm by improving various properties such as nutrient uptake, stress tolerance, resistance to antibiotics and antimicrobials and biomass production (Joshi et al. 2021; Sadiq et al. 2021; Wicaksono et al. 2022). Our experimental results showed that most of the sessile cells in Consortium 2 were in an active state, as higher ATP proportions were measured when compared to Consortium 1 (Fig. 6A). Moreover, statistical analysis demonstrated that differences in the ATP concentrations of C1 versus C2 were significant regardless of the presence of magnetite (Table S6). Simultaneously, higher microbial cell concentrations were present in the sessile communities of Consortium 2 compared to Consortium 1. Although our results suggest that the microbial diversity Consortium 2 led to better physiological conditions than Consortium 1, additional studies with microbial activity measurements at different time points are required to validate our hypothesis.

Among the biological attributes that positive interactions of the species that made up Consortium 2 might have provided to the sessile populations are higher acid stress tolerance and metabolic flexibility than C1. Nevertheless, metabolic flexibility may have only become evident in the presence of magnetite, as the test solution lacked components other than glucose as electron donors. Magnetite is a mixed-valent iron oxide that contains both Fe(II) and Fe(III), which determines its potential to be both oxidized and reduced (Byrne et al. 2015). Its role as a terminal electron acceptor (Fe(III) reduction) has been widely reported for microorganisms from the genus *Geobacter* and *Shewanella* (Schütz et al. 2015; Etique et al. 2016; Benaiges-Fernandez et al. 2019). Nevertheless, iron reduction is a metabolic pathway that fermenters can drive despite not requiring iron for growth or energy production (Su et al. 2020). During fermentation, microorganisms obtain energy by producing fermentative products, such as hydrogen, carbon dioxide and acetate, which can be used as electron acceptors to dispose of the electrons produced during oxidation reactions. Therefore, fermenters capable of iron reduction have an energetic advantage by using Fe(III) as an additional electron acceptor (Dobbin et al. 1999). The *Enterobacter* genus has proven capable of the fermentative reduction of Fe(III), the crystalline iron oxides hematite, goethite and ferrihydrite when growing in multispecies biofilms (Lentini et al. 2012; Parker et al. 2018). Hence, it is possible that besides engaging in glucose fermentation, evidenced by the acidification of the test solution, Consortium 2 in the presence of magnetite (C2 + M) was also using Fe(III) as an additional electron sink and consequently yielding higher ATP proportions than C2 (Fig. 6A). Moreover, the presence of an active population of *Pseudomonas*, a genus with the majority of species incapable of anaerobic fermentation, strongly suggests additional metabolic activities in Consortium 2.

The severe metal corrosion effect caused by microorganisms and deposits described in previous studies has been unanimously attributed to the accumulation of corrosive metabolites under the deposit, contributing to the redox reactions occurring at the metal surface and leading to higher corrosion rates (Suarez et al. 2019, 2022; Yang et al. 2022; Liao et al. 2023). In our study, local pH measurements at the metal-magnetite interphase in C2 + M (Table 2) confirmed acid accumulation under the magnetite. However, this acidification also occurred in the metal surfaces of C2 and C1 + M. Therefore, it is plausible to assert that the acidification on the metal surface, together with the higher ATP proportions measured in C2 + M (higher than C2), plausibly influenced by the metabolic flexibility of C2 over C1, and the evidenced increase in the metal surface roughness (Table S4) by magnetite contributed to the observed synergistic corrosion effects of magnetite and Consortium 2. Higher surface roughness represents a larger interfacial area with the corrosive environment, increasing the risk of pit formation (Evgeny et al. 2016). The increment of metal coupons surface roughness by magnetite can be attributed to its semi-conductive nature. Studies have shown that uniform corrosion rates of carbon steel coupled to magnetite in alkaline deaerated systems can be six times higher than when uncoupled (Song et al. 2018). Similarly, in acid (CO_2_ aqueous) systems, the galvanic cells between magnetite and mild steel have been demonstrated to accelerate localized corrosion, reporting pitting rates as high as 15 mm/year (Han et al. 2008). The porous and uneven distribution of the magnetite in our tests might have allowed the test solution to flow to the metal surface, electrically connecting the metal and magnetite and generating microgalvanic cells that were potentially aggravated in the presence of metabolic active acid-producing microbial cells potentially engaged in additional metabolic pathways.

Overall, our results emphasize that biofilm microbial composition drives the synergistic effects of magnetite and acid-producing bacteria on carbon steel corrosion. The environmental advantages that Consortium 2 had over Consortium 1 by being a more diverse community were related to the higher cell concentration and metabolic status of sessile cells in Consortium 2, which consequently had a more significant impact on the corrosion severity in the presence of magnetite.

## Data Availability

The 16S rRNA sequences were deposited in the National Centre for Biotechnology Information (NCBI) Sequence Read Archive (SRA) under bioproject number PRJNA1052409.
